# Does attitude towards wife beating determine infant feeding practices during diarrheal illness in sub-Saharan Africa?

**DOI:** 10.1186/s41182-021-00369-1

**Published:** 2021-10-09

**Authors:** Betregiorgis Zegeye, Nicholas Kofi Adjei, Bright Opoku Ahinkorah, Edward Kwabena Ameyaw, Abdul-Aziz Seidu, Comfort Z. Olorunsaiye, Sanni Yaya

**Affiliations:** 1 HaSET Maternal and Child Health Research Program, Shewarobit Field Office, Shewarobit, Ethiopia; 2 Department of Public Health, Policy and Systems, University of Liverpool, Liverpool, UK; 3 School of Public Health, Faculty of Health, University of Technology Sydney, Sydney, Australia; 4 Department of Population and Health, University of Cape Coast, Cape Coast, Ghana; 5 College of Public Health, Medical and Veterinary Sciences, James Cook University, Townsville, QLD Australia; 6 Department of Public Health, Arcadia University, Glenside, PA USA; 7 School of International Development and Global Studies, University of Ottawa, Ottawa, ON Canada; 8 The George Institute for Global Health, Imperial College London, London, UK

**Keywords:** Wife beating attitude, Women, Child feeding, Diarrhea, Sub-Saharan Africa

## Abstract

**Background:**

Inappropriate feeding practices of children during illness remains a public health problem globally, particularly in sub-Saharan Africa (SSA). One strategy to improve child health outcomes is through women empowerment—measured by wife beating attitude. However, the role of attitude towards wife beating in child feeding practices has not been comprehensively studied. Therefore, we investigated the association between women's attitude towards wife beating and child feeding practices during childhood diarrhea in 28 countries in SSA.

**Methods:**

We analyzed data from the Demographic and Health Survey on 40,720 children under 5 years. Bivariate and multivariate binary logistic regression analyses were applied to assess the association between women's attitude towards wife beating and child feeding practices. The results were presented using adjusted odds ratio (aOR) with 95% confidence intervals (CIs).

**Results:**

The pooled results showed that appropriate feeding practices during diarrheal illness among under-five children was 9.3% in SSA, varying from 0.4% in Burkina Faso to 21.1% in Kenya. Regarding regional coverage, the highest coverage was observed in Central Africa (9.3%) followed by East Africa (5.5%), Southern Africa (4.8%), and West Africa (4.2%). Women who disagreed with wife-beating practices had higher odds of proper child feeding practices during childhood diarrhea compared to those who justified wife-beating practices (aOR = 2.02, 95% CI; 1.17–3.48).

**Conclusion:**

The findings suggest that women’s disagreement with wife beating is strongly associated with proper child feeding practices during diarrheal illness in SSA. Proactive measures and interventions designed to change attitudes towards wife-beating practices are crucial to improving proper feeding practices in SSA.

## Background

Globally, under-five mortality declined by 59% from 93 deaths per 1000 live birth in 1990 to 38 deaths per 1000 live birth in 2019 [1]. In 2019, it was estimated that about 5.2 million under-five children died [1], and nearly 82% of these deaths occurred in sub-Saharan Africa (SSA) (53%) and South Asia (27%) [1].

Diarrhea is the fifth leading cause of death in children [2], accounting for approximately 8% of all deaths in children under 5 years in the world [3], next to preterm birth complication (18%), pneumonia (15%), intrapartum related events (13%) and congenital anomalies (9%), respectively [4]. It is estimated that a large proportion of diarrhea-related under-five morbidity and mortality occur in low- and middle-income countries [2], particularly in SSA [2, 4], where about half of the 616 million population used unimproved sanitation facilities [5].

Children’s nutritional status declines speedily during and after common childhood illnesses, including diarrhea [6, 7], unless extra nutrient requirements related to the illness or convalescence are provided [6–8]. To reduce diarrheal disease-related mortality and morbidity, the World Health Organization (WHO) and United Nations International Children's Emergency Fund (UNICEF) outlined a seven-point action plan for comprehensive diarrhea control by 2009 [9]. During a diarrheal episode, fluid replacement, sustained feeding, and increasing suitable fluids in the home are the cornerstones of the therapy package [9, 10]. Hence, a sick child needs breast milk and food frequently during and after illness to limit weight loss [11, 12], and aid speedy recovery [11–14]. Meanwhile, there is evidence that demographic factors and socioeconomic conditions of women may play a significant role in child feeding practices during diarrheal episodes [6, 15, 16].

Both child survival (Sustainable Development Goal (SDG-3) and women’s empowerment (SDG-5) are important global health and development priorities [17]. Ending infant and under-five mortality by 2030 is one of the sustainable development goals adopted by the United Nations in 2015 [18]. Women empowerment is one of the recently expanding strategies to promote child health and reduce childhood morbidity and mortality [17]. However, it is a multidimensional construct and may have different indicators such as economic, education, health, governance, and media [19]. One strategy to improve child health outcomes is women empowerment and women's attitude towards wife beating is an important indicator of women empowerment [21], which has been linked with maternal [22, 23] and child health outcomes [24].

In African countries, wife-beating practices are generally a common type of intimate partner violence practice, which is again perpetuated by justification or acceptance of wife-beating practices such as community norms [25, 26].

Women’s attitude towards wife-beating practices may affect child health by two mechanisms. One hypothesis is that acceptance of wife beating by women can in itself perpetuate wife-beating practices or intimate partner violence [26, 27], that may have negative consequences on their physical, mental and social health, as well as their children's health [23, 28, 29]. Second, women’s attitude towards wife beating may be associated with utilization of maternal healthcare services which may indirectly affect a child's health. For example, several studies in low-and middle-income countries have shown a relationship between wife beating attitude on maternal healthcare services [23, 30–32]. Meanwhile, there is evidence that utilization of maternal healthcare services may  influence a child's health [33–35].

While few small-scale studies have investigated child feeding practices during diarrheal illness [6, 15, 36], no study has assessed the association between women's attitude towards wife beating and child feeding practices in SSA. Therefore, this present study aimed to examine the association between women's attitude towards wife beating and feeding practice during diarrheal illness among children under five years in SSA.

## Methods

### Data source and sampling procedure

We extracted data from the  Demographic and Health Surveys (DHSs) of 28 countries in SSA for this study. The DHS is a nationally representative survey aimed at obtaining information on several demographic and health indicators, including child feeding practice. The survey is conducted across several low- and middle-income countries with financial and technical support from the United States Aid for Internal Development (USAID) and Inner-City Fund (ICF). The DHS applied a stratified two-stage cluster sampling technique. In the first stage, the primary sampling unit (PSU), which is also called enumeration areas (EAs) were selected using probability proportional to size (PPS) and the second stage involved household sampling (usually 25–30 households per cluster) using equal probability systematic sampling technique [37].

We included 28 sub-Saharan African countries for this study from the kids recode (KR) file. We limited our analyses to only married women because the independent variable (women’s attitude towards wife beating) is applicable to only married women [38]. The countries were selected if the survey was conducted between 2010 and 2020, and outcome and explanatory variables were available. The data are freely available at: https://dhsprogram.com/data/dataset_admin/login_main.cfm) [39]. The sample for the final analysis after exclusions was  40,720. Details about selected countries, year of survey and sample are shown in Table 1 below.

**Table 1:** List of studied countries, year of survey and sampled population

Country	Year of survey	Sample population	
		Weighted number	Weighted percent
Angola	2015/2016	1398	3.43
Burkina Faso	2010	1958	4.81
Benin	2017/2018	1247	3.06
Burundi	2016/2017	2355	5.78
Democratic Republic of Congo	2013/2014	2436	5.98
Congo	2011/2012	1196	2.94
Cote d’Ivoire	2011/2012	1079	2.65
Cameroon	2018/2019	880	2.16
Ethiopia	2016	1024	2.51
Gabon	2012	705	1.73
Ghana	2014	565	1.40
Gambia	2013	1220	3.00
Guinea	2018	958	2.35
Kenya	2014	2506	6.15
Comoros	2012	443	1.09
Liberia	2019/2020	651	1.60
Lesotho	2014	267	0.66
Mali	2018	1534	3.77
Malawi	2015/2016	2815	6.91
Nigeria	2018	3799	9.33
Rwanda	2014/2015	710	1.74
Sierra Leone	2019	510	1.25
Senegal	2010/2011	2054	5.04
Chad	2014/2015	3092	7.60
Togo	2013/2014	949	2.33
Uganda	2016	2487	6.11
Zambia	2018	1078	2.65
Zimbabwe	2015	804	1.97
Total		40,720	100.00

### Study variables

#### Outcome variable

The outcome variable was feeding practices of children during a recent diarrheal episode. Feeding practices were assessed by interviewing mothers with children under age 5 with diarrhea in the 2 weeks preceding the survey on the amount of food given during diarrhea. If the child had received foods during diarrhea more than usual [13, 14], we classified it as appropriate feeding practice and coded it as “yes”, otherwise it was coded as “no” [13, 14].

#### Explanatory variable

The explanatory variable for this study was women's attitude towards wife beating. In the DHS, women were asked five questions to measure their attitude towards wife-beating practices. The questions posed were for example “Do you agree that a husband is justified in hitting or beating his wife when she burns food”? “Do you agree that a husband is justified in hitting or beating his wife when she argues with him”? “Do you agree that a husband is justified in hitting or beating his wife when she is going out without telling him”? “Do you agree that a husband is justified in hitting or beating his wife when she neglects the children”? “Do you agree that a husband is justified in hitting or beating his wife when she refuses to have sex with him”? Responses were coded “0” if a woman responded in the affirmative (accepted) to at least one of the five above-mentioned wife beating questions [38, 40, 41], otherwise coded “1” if a woman did not justify or disagreed with wife-beating practices for all five above-mentioned reasons.

#### Confounding variables

In line with prior studies [6, 15, 36, 42–44], the following confounders were considered: women’s age in years (15–19, 20–24, 25–29, 30–34, 35–39, 40–49), women’s educational level (no formal education, primary school, secondary school, and higher), husband educational level (no formal education, primary school, secondary school, and higher), place of residence (urban, rural), religion (Christian, others), parity (1–2, 3–4, and 5 +), family size (< 5, 5 +) and currently employed (no, yes). Other included covariates were media exposure (no, yes), distance to health facility (not a big problem, a big problem) and wealth quintiles (quintile 1, quintile 2, quintile 3, quintile 4, and quintile 5). The DHS wealth index was computed using durable goods, household characteristics and basic services following the methodology explained elsewhere [45], and we followed same procedure.

### Statistical analysis

First, summary descriptive measures were estimated and presented using tables and bar charts. Second, bivariate logistic regression was conducted to estimate the crude effect of the explanatory and control variables on the outcome variable (child feeding practices), to select candidate variables for the multivariable logistic regression analysis using a *p*-value less than or equal to 0.05. This was followed by a multicollinearity test on all the explanatory or control variables that had significant associations in the bivariate analysis. We found no evidence of collinearity among the explanatory or control variables (Mean VIF = 2.35, Min VIF = 1.13, Max VIF = 7.24). Variance inflation factor (VIF) values less than 10 are tolerable [46, 47].

Third, a multivariable logistic regression was fitted to examine the relationship between child feeding practices and the explanatory/confounding variables. The results were presented using adjusted odds ratio (aOR) with 95% confidence intervals (CIs). The goodness-of-fit of the regression model was assessed using Hosmer–Lemeshow test [48]. The model showed a better fit (*p* = 0.3071). Data processing and analysis were performed using Stata, V.14.2 (Stata Corp, College Station, Texas, USA). The “svyset” command in STATA was used to account for the complex survey design including weight, cluster, and strata.

## Results

### Background characteristics of respondents

The study included 40,720 mothers of under-five children. Table 3 shows results on the characteristics of respondents. About 6.3% of respondents were adolescents (15–19 years) and 40.1% were rural residents. Nearly 29.8% and 25.1% of the respondents had no formal education and were not currently employed, respectively. More than half (53.9%) of the respondents had a big problem reaching either a health center or hospital. Approximately 68.5% of currently married women disagreed with all five reasons of wife-beating practices.

### Practice of appropriate child feeding during diarrhea

**Fig. 1:**
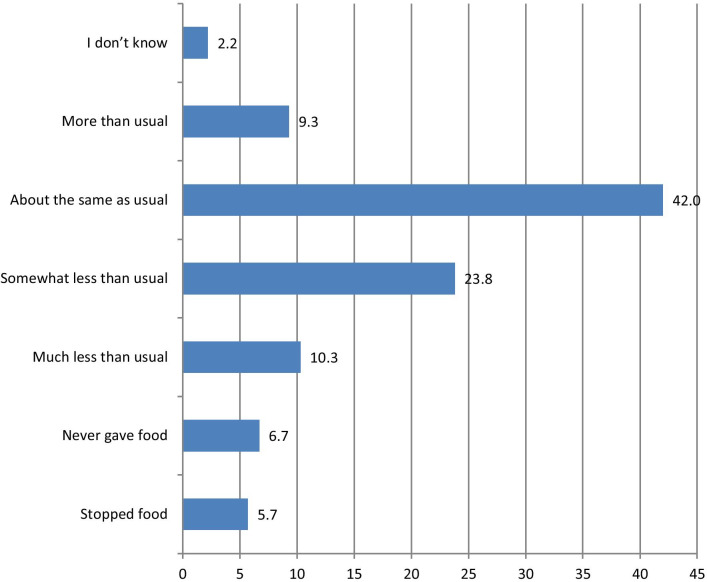
Practice of appropriate feeding during diarrhea among under-five age children: evidence from the DHSs of 28 countries in SSA

The pooled analysis shows that about 9.3% of under-five children were offered more foods during diarrhea. However, about 42.0% and 34.1% were offered foods the same as usual and less than usual, respectively. Approximately 6.7% of under-five children were never given foods, while about 5.7% stopped foods during diarrhea (Fig. 1).

Table 2 shows results of feeding practices during childhood diarrhea in SSA.  We observed variations in the sub-regional coverage of proper child feeding practices during diarrhea. The highest and lowest coverage were in Central (9.3%) and West Africa (4.2%), respectively.

**Table 2:** Sub-Saharan African regions proper feeding practice among under-five children from married women: evidence from the DHSs of 28 countries in SSA

Sub-Saharan African regions	Included countries	Pooled sub-regional coverage
West Africa	Burkina Faso	4.2% (95% CI; 3.3–5.4%)
	Benin	
	Côte d’Ivoire	
	Ghana	
	Gambia	
	Guinea	
	Liberia	
	Mali	
	Nigeria	
	Sierra Leone	
	Senegal	
	Togo	
Central Africa	Angola	9.3% (95% CI; 6.4–13.4%)
	Congo	
	Congo Democratic Republic	
	Cameroon	
	Gabon	
	Chad	
Southern Africa	Lesotho	4.8% (95% CI; 2.7–8.4%)
East Africa	Burundi	5.5% (95% CI; 4.5–6.8%)
	Ethiopia	
	Kenya	
	Comoros	
	Malawi	
	Rwanda	
	Uganda	
	Zambia	
	Zimbabwe	

**Fig. 2:**
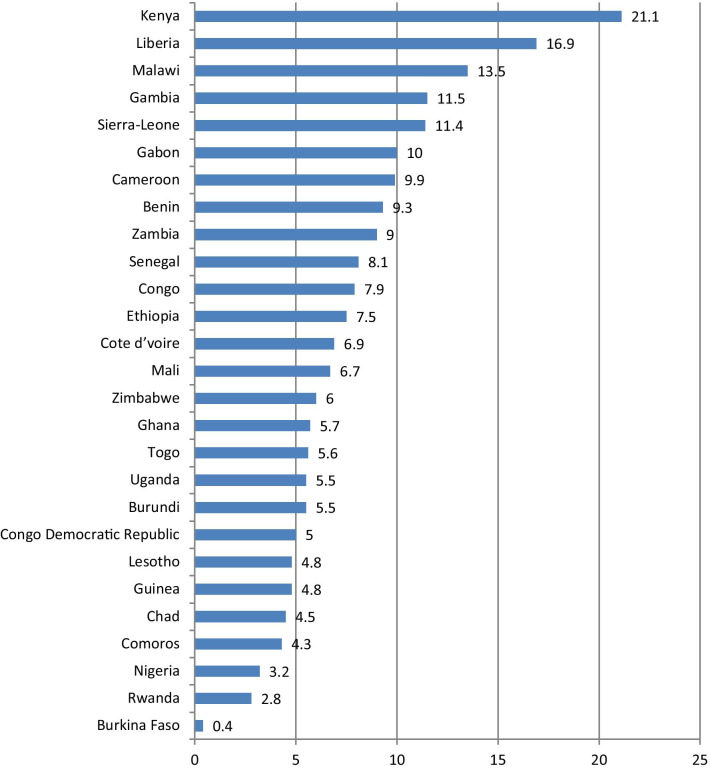
Coverage of proper child feeding practice during diarrheal among children of under-5 years across countries: evidence from the DHSs of 28 countries in SSA

Regarding country-specific distribution, the lowest prevalence of proper child feeding practice during diarrhea was observed in Burkina Faso (0.4%), Rwanda (2.8%) and Nigeria (3.2%), while the highest was observed in Kenya (21.1%), Liberia (16.9%) and Malawi (13.5%) (Fig. 2).

### Distribution of feeding practices by explanatory/confounding variables

Table 3 shows the distribution of child feeding practices during diarrhea by explanatory/confounding variables and subgroups. Child feeding practices varied from 5.8% among under-five children who had adolescent mothers (15–19 years) to 16.3% among mothers who were 45–49 years. We also observed that proper child feeding during childhood diarrhea varied from 5.5% among under-five children from currently married women who disagreed with wife beating to 11.5% among under-five children from currently married women who agreed with or justified wife beating. Proper child feeding practices during diarrhea also varied significantly by economic status, ranging from 3.3% among the poorest households to 17.0% among the richest households.

**Table 3:** Socioeconomic characteristics of respondents and distribution of feeding practice by explanatory variables: evidence from 28 SSA DHSs

Variable	Number (weighted %)	Proper feeding (weighted %)	
		Yes	No
*Justification of Wife beating *			
Yes	140,831 (31.49)	5.5	94.5
No	132,775 (68.51)	11.5	88.5
*Women’s age*			
15–19	13,518 (6.26)	5.8	94.2
20–24	60,698 (25.00)	6.5	93.5
25–29	79,024 (26.32)	10.4	89.6
30–34	61,594 (18.94)	9.1	90.9
35–39	42,664 (13.48)	10.9	89.1
40–49	25,451 (10.00)	16.3	83.7
*Women’s educational level*			
No formal education	125,770 (29.8)	5.5	94.5
Primary school	90,483 (41.19)	11.7	88.3
Secondary school	58,106 (26.51)	10.2	89.8
Higher	8,574 (2.50)	1.7	98.3
*Husband educational level*			
No formal education	102,438 (14.73)	4.9	95.1
Primary school	71,187 (32.96)	8.1	91.9
Secondary school	74,211 (46.23)	11.2	88.8
Higher	17,725 (6.07)	7.6	92.4
*Wealth index*			
Poorest	74,372 (20.59)	3.3	96.7
Poorer	63,546 (24.07)	3.7	96.3
Middle	55,822 (22.62)	12.7	87.3
Richer	48,583 (18.00)	17.0	83.0
Richest	40,626 (14.72)	10.2	89.8
*Place of residence*			
Urban	81,321 (59.95)	12.9	87.1
Rural	201,628 (40.05)	3.4	96.6
*Media exposure*			
No	110,642 (32.17)	4.9	95.1
Yes	171,768 (67.83)	11.0	89.0
*Religion*			
Christian	154,821 (92.99)	9.8	90.2
Others	127,902 (7.01)	3.4	96.6
*Parity*			
1–2	84,204 (25.34)	7.2	92.8
3–4	91,484 (33.95)	8.7	91.3
5 +	107,261 (40.71)	11.4	88.6
*Family size*	Freq		
< 5	70,472 (25.77)	5.9	94.1
5 +	212,477 (74.23)	10.6	89.4
*Distance to health facility*			
Not big problem	151,692 (46.07)	9.2	90.8
A big problem	110,507 (53.93)	9.4	90.6
*Currently employed*			
No	100,396 (25.05)	8.5	91.5
Yes	172,967 (74.95)	9.6	90.4

### Bivariate and multivariable binary logistic regression results

Table 4 shows the results of the bivariate and multivariate binary logistic regression analysis. We found that women's attitude towards wife beating, women’s educational level, household wealth quintiles, place of residence, media exposure, and family size were significantly associated with child feeding practices in the bivariate logistic regression. However, in the multivariable logistic regression analysis, only women's attitude towards wife beating was significantly associated with appropriate child feeding practice. We found the odds of proper child feeding practice to be higher among women who disagreed with wife-beating practice (aOR = 2.02, 95% CI; (1.17–3.48) compared to those who agreed with or justified wife-beating practice (Table 4).

**Table 4:** Bivariate and multivariable binary logistic regression results for women's attitude towards wife beating (disagreed with wife beating) as predictor of appropriate child feeding practice during diarrhea among under-five age children: evidence from the DHSs of 28 countries in SSA

Variable	Model I		Model II	
	cOR (95% CI)	*p*-value	aOR (95% CI)	*p*-value
*Wife beating attitude*				
Agreed/justified	Ref		Ref	
Disagreed/not justified	2.21 (1.17–4.18)	**0.014**	2.02 (1.17–3.48)	**0.012**
*Women’s age*				
15–19	Ref		–	–
20–24	1.12 (0.36–3.43)	0.840	–	–
25–29	1.87 (0.63–5.49)	0.254	–	–
30–34	1.60 (0.46–5.59)	0.453	–	–
35–39	1.96 (0.50–7.72)	0.332	–	–
40–49	3.13 (0.75–13.09)	0.116	–	–
*Women’s educational level*				
No formal education	Ref		Ref	
Primary school	2.29 (1.05–4.99)	**0.036**	1.56 (0.74–3.30)	0.235
Secondary school	1.95 (0.91–4.17)	0.083	0.85 (0.30–2.37)	0.757
Higher	0.30 (0.03–2.80)	0.293	0.15 (0.01–1.71)	0.127
*Husband educational level*				
No formal education	Ref		–	–
Primary school	1.70 (0.54–5.33)	0.360	–	–
Secondary school	2.44 (0.95–6.27)	0.063	–	–
Higher	1.58 (0.36–6.78)	0.536	–	–
*Wealth quintiles*				
Quintile 1	Ref		Ref	
Quintile 2	1.10 (0.37–3.25)	0.852	0.86 (0.28–2.60)	0.794
Quintile 3	4.24 (1.51–11.90)	**0.006**	2.06 (0.61–6.90)	0.239
Quintile 4	5.96 (1.88–18.85)	**0.002**	2.53 (0.62–10.28)	0.191
Quintile 5	3.30 (0.99–11.01)	0.051	1.69 (0.36–7.76)	0.497
*Place of residence*				
Urban	Ref		Ref	
Rural	0.23 (0.11–0.49)	**0.000**	0.45 (0.19–1.08)	0.075
*Media exposure*				
No	Ref		Ref	
Yes	2.40 (1.24–4.64)	**0.009**	1.10 (0.58–2.07)	0.759
*Religion*				
Christian	Ref		–	–
Others	0.32 (0.08–1.25)	0.105	–	–
*Parity*			–	–
1–2	Ref		–	–
3–4	1.22 (0.66–2.24)	0.508	–	–
5 +	1.64 (0.70–3.84)	0.250	–	–
*Family size*				
< 5	Ref		Ref	
5 +	1.91 (1.03–3.53)	**0.039**	1.58 (0.88–2.85)	0.125
*Distance to health facility*				
Not big problem	Ref		–	–
A big problem	1.02 (0.47–2.24)	0.945	–	–
*Currently employed*			–	–
No	Ref		–	–
Yes	1.13 (0.55–2.32)	0.730	–	–

*Ref* reference, *CI* confidence interval, *cOR* crude odds ratio, aOR adjusted odds ratio, (–) not included variable in model II (adjusted model) due to non-significant results in model I (unadjusted model)

Table 5 shows country-specific unadjusted and adjusted results for association between women’s attitude towards wife beating and proper child feeding practice during childhood diarrhea. We found higher odds of proper child feeding practice during diarrheal illness among married women who disagreed with wife beating in Guinea (aOR = 1.98, 95% CI; 1.07–3.66) compared to those who were agreed or justified with wife beating. The odds of appropriate child feeding practice during diarrheal illness among married women who disagreed with wife beating was lower in Benin (aOR = 0.36, 95% CI; 0.24–0.56) and Cameroon (aOR = 0.61, 95% CI; 0.39–0.94) as compared those who justified wife beating (Table 5).

**Table 5:** Bivariate and multivariable logistic regression outputs for predictors of proper child feeding practice during diarrheal among under-five age children by country: evidence from the DHSs of 28 countries in SSA

Country	Model I		Model II	
	cOR [95% CI]	*p*-value	aOR [95% CI]	*p*-value
Angola	1.30 (0.85–1.96)	0.213	1.22 (0.79–1.87)	0.355
Burkina Faso	0.85 (0.55–1.32)	0.484	0.75 (0.47–1.18)	0.219
Benin	0.34 (0.22–0.51)	**0.000**	0.36 (0.24–0.56)	**0.000**
Burundi	0.93 (0.64–1.37)	0.745	0.95 (0.65–1.39)	0.806
Democratic Republic of Congo	0.98 (0.61–1.58)	0.945	1.06 (0.65–1.72)	0.809
Congo	0.85 (0.53–1.36)	0.509	0.91 (0.56–1.46)	0.698
Cot’ devoir	0.70 (0.44–1.13)	0.155	0.70 (0.43–1.13)	0.148
Cameroon	0.66 (0.43–1.00)	0.052	0.61 (0.39–0.94)	**0.028**
Ethiopia	0.71 (0.40–1.28)	0.262	0.67 (0.36–1.23)	0.203
Gabon	1.24 (0.75–2.03)	0.391	1.25 (0.76–2.07)	0.370
Ghana	2.01 (0.92–4.39)	0.080	1.90 (0.82–4.39)	0.132
Gambia	0.80 (0.53–1.20)	0.295	0.86 (0.55–1.34)	0.524
Guinea	1.95 (1.07–3.54)	**0.028**	1.98 (1.07–3.66)	**0.029**
Kenya	1.15 (0.86–1.54)	0.316	1.02 (0.75–1.39)	0.862
Comoros	4.08 (1.16–14.30)	**0.028**	3.48 (0.97–12.44)	0.055
Liberia	0.71 (0.45–1.11)	0.141	0.68 (0.42–1.09)	0.113
Lesotho	2.34 (0.72–7.56)	0.154	2.87 (0.78–10.49)	0.109
Mali	0.75 (0.40–1.39)	0.365	0.82 (0.43–1.55)	0.553
Malawi	1.05 (0.79–1.39)	0.713	1.05 (0.79–1.39)	0.716
Nigeria	1.18 (0.85–1.65)	0.313	0.99 (0.69–1.41)	0.962
Rwanda	0.84 (0.33–2.11)	0.714	0.87 (0.33–2.27)	0.783
Sierra Leone	0.73 (0.39–1.36)	0.332	0.74 (0.39–1.38)	0.351
Senegal	0.78 (0.53–1.13)	0.196	0.70 (0.47–1.03)	0.077
Chad	0.90 (0.57–1.41)	0.658	0.91 (0.56–1.46)	0.704
Togo	1.27 (0.73–2.20)	0.393	1.49 (0.84–2.67)	0.170
Uganda	1.23 (0.87–1.74)	0.230	1.02 (0.72–1.46)	0.874
Zambia	1.80 (1.16–2.77)	**0.008**	1.48 (0.93–2.34)	0.093
Zimbabwe	1.51 (0.82–2.78)	0.181	1.59 (0.85–2.96)	0.144

*CI* confidence interval, *cOR* crude odds ratio, *aOR* adjusted odds ratio, Model I is a model that contain unadjusted results, Model II is a model with adjusted results

## Discussion

In this study, we examined the association between women's attitude towards wife beating and appropriate child feeding practices during diarrhea among under-five children, using nationally representative surveys from 28 countries in SSA. Overall, our study showed that proper feeding practices during diarrhea among under-five children was 9.3% (95% CI; 6.4–13.4%) in SSA, varying from 0.4% in Burkina Faso to 21.1% in Kenya. We further found regional differences, where the highest coverage was observed in Central Africa (9.3%) followed by East Africa (5.5%), Southern Africa (4.8%), and West Africa (4.2%).

The pooled results from this study were found to be lower than a study conducted in Ethiopia (15.4%) [36]. The variations might be due to differences in methodology [36], as the previous study was based on a single country with a smaller sample size [36]. Furthermore, variations in societal infant feeding habits and utilization of healthcare services by women may partly explaine the differences in infant feeding across countries [49, 50]. For instance, a recent study in three African countries showed that Burkina Faso has less access to and utilization of maternal healthcare services which results in variation in infant feeding practices [49]. Another justification might be due to differences in educational level. In same study, the authors noted that Burkina Faso has generally lower educational level compared to Uganda and South Africa, and this may account for the differences in maternal knowledge about infant feeding practices [49]. Maternal knowledge has been shown to be  a significant factor for infant feeding practices,  but the rate is lower in Burkina Faso as documented by a study in Boucle du Mouhoun, Burkina Faso [51].

The country-specific result showed, lower odds of proper child feeding practice among married women in Benin and Cameroon who agreed with wife-beating practices. We note that this finding is unusual, and thus recommend more quantitative and qualitative studies to produce further evidence about women’s attitude towards wife-beating practices and its relationship with child health including child feeding practices during childhood diarrhea.

Our study showed that married women with children under five years who disagreed with wife-beating practices were more likely to engage in appropriate child feeding practices during diarrhea than those who agreed with or justified wife beating. A possible explanation for this phenomenon may be that women who disagreed with wife beating may have higher socioeconomic status [52] and a higher degree of decision-making power [41, 42, 52–54]. Moreover, there is strong evidence that education, for instance, is a strong predictor of wife beating attitude [55].

Another plausible explanation for the higher odds of appropriate feeding practices among women with children under 5 years old who disagreed with wife beating might be related to better utilization of health services [22, 56], including ANC and skilled delivery services as compared to women who agreed or justified wife beating [22, 56]. Better childcare and health seeking behavior for child health services have been reported among women who utilize maternal health services and visit health institutions [16, 57, 58]. Previous studies from SSA noted that while gender-based violence persists in the region [59], accepting wife beating as a healthy norm appears to be decreasing [60, 61], perhaps due to the increasing advocacy in developmental programs [60, 61] and activities targeting women empowerment over the past decades [59, 60]. Thus, strengthening similar and other bespoke interventions may help to reduce the acceptance of wife beating [40–42, 60, 61]. This may further help to improve child health outcomes and practices including proper feeding practices.

### Strengths and limitations of the study

The use of large nationally representative sample and multiple countries with a large sample size are the key strengths of this study. However, a cause–effect relationship cannot be established because of the cross-sectional nature of the study. Second, the DHS relied on self-reported data which may be prone to recall bias. Third, we limited our study to only married women and 28 sub-Saharan African countries, thus generalization of the findings may not be possible. Lastly, due to data availability and constraints, we used surveys that were conducted at different time points in the selected countries.

## Conclusion

The findings suggest that women’s disagreement with wife beating is strongly associated with proper child feeding practices during diarrheal diseases among children under-five in SSA. Thus, proactive policies and interventions designed to change attitudes towards wife-beating practices are crucial to improving proper feeding practices in the region. Measures including socioeconomic empowerment engagement with religious and community leaders and awareness creation about the negative consequences of wife-beating may help reduce acceptance of wife-beating practices [26, 60].

## Data Availability

Data for this study were sourced from Demographic and Health surveys (DHS) and available here: http://dhsprogram.com/data/available-datasets.cfm.
